# Vaccination information, motivations, and barriers in the context of meningococcal serogroup A conjugate vaccine introduction: A qualitative assessment among caregivers in Burkina Faso, 2018

**DOI:** 10.1016/j.vaccine.2021.09.038

**Published:** 2021-10-15

**Authors:** Brooke Noel Aksnes, Jenny A. Walldorf, Sylvain F. Nkwenkeu, Robert L. Zoma, Imran Mirza, Felix Tarbangdo, Soukeynatou Fall, Sansan Hien, Cesaire Ky, Ludovic Kambou, Alpha Oumar Diallo, Flavien H. Aké, Cynthia Hatcher, Jaymin C. Patel, Ryan T. Novak, Terri B. Hyde, Isaïe Medah, Heidi M. Soeters, Mohamed F. Jalloh

**Affiliations:** a Centers for Disease Control and Prevention, Atlanta, GA, 30329, USA; b UNICEF Ouagadougou, Burkina Faso; c Institut National de Statistique et Démographie, Ouagadougou, Burkina Faso; d UNICEF, New York, NY 10017, USA; e Davycas International, Ouagadougou, Burkina Faso; f Ministère de la Santé, Ouagadougou, Burkina Faso

**Keywords:** Burkina Faso, Meningococcal serogroup A conjugate vaccine, Measles vaccine, Immunization schedule, Vaccine introduction, Vaccination challenges

## Abstract

**Background:**

In March 2017, Burkina Faso introduced meningococcal serogroup A conjugate vaccine (MACV) into the Expanded Programme on Immunization. MACV is administered to children aged 15–18 months, concomitantly with the second dose of measles-containing vaccine (MCV2). One year after MACV introduction, we assessed the sources and content of immunization information available to caregivers and explored motivations and barriers that influence their decision to seek MACV for their children.

**Methods:**

Twenty-four focus group discussions (FGDs) were conducted with caregivers of children eligible for MACV and MCV2. Data collection occurred in February–March 2018 in four purposively selected districts, each from a separate geographic region; within each district, caregivers were stratified into groups based on whether their children were unvaccinated or vaccinated with MACV. FGDs were recorded and transcribed. Transcripts were coded and analyzed using qualitative content analysis.

**Results:**

We identified many different sources and content of information about MACV and MCV2 available to caregivers. Healthcare workers were most commonly cited as the main sources of information; caregivers also received information from other caregivers in the community. Caregivers’ motivations to seek MACV for their children were driven by personal awareness, engagements with trusted messengers, and perceived protective benefits of MACV against meningitis. Barriers to MACV and MCV2 uptake were linked to the unavailability of vaccines, immunization personnel not providing doses, knowledge gaps about the 15–18 month visit, practical constraints, past negative experiences, sociocultural influences, and misinformation, including misunderstanding about the need for MCV2.

**Conclusions:**

MACV and MCV2 uptake may be enhanced by addressing vaccination barriers and effectively communicating vaccination information and benefits through trusted messengers such as healthcare workers and other caregivers in the community. Educating healthcare workers to avoid withholding vaccines, likely due to fear of wastage, may help reduce missed opportunities for vaccination.

## Highlights

• Caregivers were motivated to vaccinate their children against measles and meningitis.
• Motivations were driven by vaccine communication and perceived disease protection.
• Health workers were trusted sources of vaccine information.
• Barriers included vaccine unavailability, knowledge gaps, and practical constraints.
• Vaccine uptake could be enhanced by using trusted messengers and addressing barriers.

## 1 Introduction

The meningitis belt of sub-Saharan Africa—including over 400 million people in 26 countries stretching from Senegal to Ethiopia—experiences high endemic rates of meningitis, annual seasonal outbreaks, and explosive epidemics occurring every 5–10 years [1]. Prior to the introduction of the meningococcal serogroup A conjugate vaccine (MACV, MenAfriVac®), approximately 90% of all meningitis cases detected in the region were attributable to *Neisseria meningitidis* serogroup A [2]. Beginning in 2010, MACV was progressively rolled out, using mass vaccination campaigns targeting persons aged 1–29 years [3]. The campaigns received high community acceptance and achieved a regional aggregate administrative coverage of 98% in the targeted population by 2018 [3], [4]. Following the mass campaigns, the incidence of serogroup A meningitis cases and outbreaks sharply declined [5], [6], [7]. To ensure long-term disease suppression, the World Health Organization (WHO) recommended that meningitis belt countries introduce one dose of MACV into the routine childhood Expanded Programme on Immunization (EPI) schedule at 9–18 months of age within 1–5 years after mass campaign completion, along with a one-time catch-up campaign for children born in the interim [8].

Burkina Faso completed the initial MACV mass campaign in 2010, conducted a catch-up campaign for children aged 1–6 years in November 2016, and in March 2017, became the fourth country in the meningitis belt of sub-Saharan Africa to introduce MACV into the routine EPI schedule [3]. In Burkina Faso, MACV is given to children aged 15–18 months together with a second dose of measles-containing vaccine (MCV2) at the same immunization visit. Both vaccines are supplied in 10-dose vials. Given the high community acceptance of MACV during campaigns, there were expectations that the introduction of MACV into the routine EPI schedule would encourage caregivers to bring their children for vaccination, and in turn, improve uptake of MCV2 among children receiving the two vaccines at the same visit [9].

MCV2 was introduced in Burkina Faso in October 2013. National estimated MCV2 coverage was 17% in 2014 but increased to 50% in 2015 and reached 71% by 2018 [10]. Like other low- and middle-income countries (LMICs), there is a high dropout rate between the first dose of MCV (MCV1) and MCV2 (i.e., a high proportion of children start but do not complete the vaccine series). For example, in 2018, MCV1 coverage in Burkina Faso was 88% compared to MCV2 coverage of 71% [10]. Suboptimal coverage is commonly observed with other vaccines given during the second year of life in many countries [11], [12]. In 2018, one year after MACV introduction in Burkina Faso, a nationwide household cluster survey found that national MACV coverage via the EPI was 58% among children aged 18–26 months. A small yet significant increase (4.5%) in MCV2 coverage was recorded following MACV introduction, though could not be distinguished from the expected increase in MCV2 coverage over time; and MACV and MCV2 co-administration was common [9].

An interplay of supply- and demand-side factors impact access to and acceptance of childhood vaccines in LMIC contexts [13]. These include operational and logistical factors in effectively transporting, managing, and storing vaccines; vaccine supply shortages; inadequate healthcare worker staffing; sociocultural influences such as religious beliefs; and parental knowledge and normative attitudes [13]. Previous qualitative assessments in Burkina Faso found that the important behavioral drivers of vaccination were parental understanding of childhood diseases, knowledge of the immunization schedule, logistical and practical constraints, and past experiences with immunization services, including adverse events following immunization and interactions with healthcare workers [14], [15], [16], [17], [18].

To better understand various aspects of MACV introduction into Burkina Faso’s EPI schedule, we conducted a qualitative assessment in 2018. The first component of the assessment focused on gathering health workers’ perceptions and challenges through in-depth interviews; the results have been described elsewhere [19]. In summary, immunization staff commonly perceived MACV introduction as a source of motivation for caregivers to bring their children for the 15–18-month visit. The assessment also identified barriers around supply, health systems, and demand. A major barrier was the reluctance of health workers to open multi-dose vials unless there were enough eligible children to vaccinate, which was tied to a misguided effort to avoid vaccine wastage. In this report, we describe the second component of the qualitative assessment that focused on the perspectives of caregivers of children eligible for MACV and MCV2. Specifically, we aimed to understand the sources and content of information available on MACV and MCV2 for caregivers in Burkina Faso and to explore what drives the decision to seek MACV for their children. This study adds new understanding, in addition to reinforcing the existing information around motivations and barriers to seeking immunization services—evidence that is essential to further optimize vaccination coverage in Burkina Faso.

## 2 Methods

We used an exploratory qualitative design to evaluate caregivers’ perceptions and obtain their feedback on the introduction of MACV in Burkina Faso. The assessment comprised 24 focus group discussions (FGDs) that were conducted between February and March 2018 in four districts. We have reported the methods and results of the assessment in accordance with the guidelines for Consolidated Criteria for Reporting Qualitative Studies [20].

### 2.1 Sampling

Table 1 describes the districts and categories of primary caregivers selected for inclusion in the assessment. We purposively selected one district each from four geographic regions (Nord, Centre, Centre-Est, and Cascades) to reflect variations in administrative vaccination coverage and to account for rural–urban demographic characteristics. Koupela (Centre-Est; rural) and Baskuy (Centre; urban) districts were selected because of their low administrative MCV2 coverage (<50%). Mangodara (Cascades; rural) and Ouahigouya (Nord; urban) were selected due to their high MCV2 coverage (>90%). Within districts, communities were selected via convenience sampling. We recruited primary caregivers of children who were eligible to receive MACV and MCV2 (i.e., 15–26 months old). Primary caregiver was defined as the person responsible to take the child for scheduled vaccination visits. Caregivers were recruited with the help of community health workers. In addition, caregivers were recruited through snowball sampling whereby identified caregivers helped data collection teams identify other eligible caregivers who were then approached for eligibility screening and consent.

**Table 1:** Selection criteria for focus group discussions with caregivers, qualitative assessment, Burkina Faso, 2018.

Region (district)	Administrative MCV2 coverage	Setting	Number of focus groups of caregivers with MACV vaccinated children	Number of focus groups of caregivers with MACV unvaccinated children	Total number of focus groups
Cascades (Mangodara)	High (>90%)	Rural	3	3	6
Nord (Ouahigouya)	High (>90%)	Urban	3	3	6
Centre-Est (Koupela)	Low (<50%)	Rural	3	3	6
Centre (Baskuy)	Low (<50%)	Urban	3	3	6
Total number of focus groups			12	12	24

*Abbreviations:* MACV, meningococcal serogroup A conjugate vaccine; MCV2, second dose of measles-containing vaccine.

To capture the variation in MACV vaccination behaviors, we stratified caregivers in each district into two groups: 1) those whose children had received MACV (“vaccinated group”) and 2) those whose children were age-eligible but had not received MACV (“unvaccinated group”). A caregiver’s child’s vaccination status was ascertained using the home-based card record when available and using caregiver recall when the card was unavailable.

### 2.2 Development of FGD guides and training

A team of technical experts from the Burkina Faso Ministry of Health, Davycas International (a local non-governmental organization), the United Nations Children’s Fund (UNICEF), and the U.S. Centers for Disease Control and Prevention (CDC) developed two overlapping but distinct FGD guides (Supplemental Material). Separate guides were developed for the FGDs with caregivers whose children were vaccinated with MACV and for the FGDs planned with caregivers whose children were unvaccinated with MACV. Four data collectors and two team supervisors were recruited by Davycas International; all of them were nationals of Burkina Faso, were fluent in the required local languages, had experience with qualitative data collection, and possessed a bachelor’s degree in social sciences or public health.

Several of the co-authors who have experience conducting qualitative research in diverse sub-Saharan Africa contexts (MFJ, AOD, SFN, SF, SN, JAW) led the training of the data collection teams. The training of data collectors lasted one week and covered all aspects of the methodology, including the selection of respondents, informed consent, qualitative interviewing techniques, proper use of the FGD guides, data management, transcription, and quality control. In addition to practicing FGD facilitation techniques through roleplay during the training, each data collection team member pre-tested one or both FGD guides with a convenience sample of caregivers. The pre-testing informed subsequent revisions to the guides to improve the framing and sequencing of the questions and probes.

### 2.3 Data collection

The trained data collectors were assigned roles as interviewers and note-takers, with supervisors performing quality control checks with the data collection teams. FGDs were carried out in the predominant local languages in the districts. Before participating in an FGD, all recruited caregivers provided written informed consent. We audio-recorded the FGDs with the permission of the caregivers. At the end of each FGD, the data collectors and supervisors used a structured template to debrief on their observations, interview dynamics, and contextual issues. Only a few caregivers declined participation in the FGDs due to conflicting schedules and for other reasons that were not documented. The FGDs were conducted in a quiet area in the community that was jointly identified by data collectors and participating caregivers. We did not conduct repeat FGDs to follow-up on themes identified in FGDs because of resource limitations. Data saturation was partly informed by ongoing assessment of debriefing notes drafted by interviewers and note-takers after each FGD based on discussions and observations but was confirmed during the analytical phase.

### 2.4 Data processing and analysis

The data collectors simultaneously translated and transcribed the audio recordings of the FGDs they conducted. Twelve (half) of the transcripts were checked against the audio recordings by supervisors as part of the quality control. Whenever discrepancies were identified, supervisors resolved them via consultations with the respective data collectors. A web-based platform, Dedoose (www.dedoose.com), was used to organize, manage, and code the transcripts by meaning unit (a grouping of statements that convey the same central meaning). In the first part of the analysis, one analyst applied deductive codes to identify meaning units in the text. In the second part of the analysis, aiming to discover why some children were vaccinated against MACV and MCV2 while others were unvaccinated, we used a qualitative content analysis approach to organize and interpret the meaning units by sub-categories and categories. We then systematically reviewed the coded data to identify thematic explanations of MACV and MCV2 vaccination behaviors in the context of MACV introduction in Burkina Faso. Table 2 specifies the meaning units, categories and sub-categories and themes used in the analysis.

**Table 2:** Grouping of meaning units into sub-categories, categories, and themes, qualitative assessment, Burkina Faso, 2018.

Sub-categories	Categories	Themes
Vaccination reminder	Information content	Information about MACV and MCV2
Vaccination benefits		
Healthcare workers	Information sources	
Community health workers		
Mass media (TV, radio)		
Other caregivers		
Traditional and religious leaders		
Town criers		
Awareness of vaccine schedule	Awareness	Motivations to seek MACV for the child
Awareness of ‘no-cost’ for vaccines		
Trust in healthcare workers	Trusted messengers	
Distrust in healthcare workers		
Other caregivers in the community		
Severity of meningitis	Perceived benefits	
No more meningitis cases		
Protection against meningitis		
Failure to make vaccines available	Unavailability or withholding of vaccines	Barriers to the uptake of MACV and MCV2
Fear of wastage		
Multiple trips to health facilities		
Inability to pay at private clinics		
Not knowing importance of the 15–18-month visit	Knowledge gaps	
Difficult technical information		
Not knowing the vaccine schedule		
Lack of time to travel	Practical constraints	
Long wait time at healthcare center		
Inclement weather		
Harvest season		
Unpleasant experiences with healthcare workers	Past negative experiences	
Shaming women who missed visits		
Stigmatization of farming women	Sociocultural influences	
Stigma for not birth spacing		
No need for MACV if other vaccines received	Misinformation	
No need for repeat visit at 15 months		
Healthy children don’t need vaccines		
Vaccines paralyze, sterile, or kill children		

### 2.5 Ethical considerations

The assessment protocol was approved by the Ethics Committee for Health Research in Burkina Faso. In addition, the project was reviewed in accordance with CDC human research protection procedures and was determined to be a routine public health activity not requiring CDC Institutional Review Board review.

## 3 Results

Our 24 FGDs comprised of primary caregivers who were all mothers of MACV-eligible children ages 15-26 months old. Per the study design, half of the caregivers had children who were vaccinated with MACV while the other half had children who had not received MACV. In summary, we identified diverse information sources and content available for all caregivers on MACV and MCV2. Caregivers’ motivations to seek MACV for their children were mainly driven by awareness, engagement with trusted messengers and other caregivers in the community, and the perceived protective benefits of MACV. Healthcare workers were commonly cited as the main sources of information, while caregivers also received information from other caregivers and elder village women in the community. General barriers to MACV and MCV2 uptake included the unavailability of vaccines, withholding of vaccines by immunization personnel, knowledge gaps, practical constraints, sociocultural influences, and misinformation about vaccination, with caregivers of unvaccinated children additionally listing past negative experiences with healthcare workers.

### 3.1 Information about MACV and MCV2

#### 3.1.1 Information content

Caregivers reported receiving various messages reminding them to return to the health center for the 15–18-month visit, along with messages promoting the protective benefits of MACV and MCV2 against meningitis and measles, respectively, and the overall health benefits of childhood vaccination.

*“After the nine-month vaccination visit, the health workers say to return at fifteen months for the meningitis and measles vaccination.”* (Nord, vaccinated group)

*“They tell us that coming to the 15-month visit means protecting your child against diseases.”* (Nord, unvaccinated group)

#### 3.1.2 Information sources

Caregivers described receiving information about MACV from multiple and diverse sources, including healthcare workers, community health workers, mass media programs (e.g., via television or radio), other caregivers in their community, cultural and religious leaders, and town criers.

*“Health workers give immunization information to the village chief, who in turn is responsible for informing the population. The chief tells us to send the children to receive the vaccination against meningitis.”* (Cascades, unvaccinated group)

*“For those who do not regularly come to be weighed, the nurses shared leaflets in all households. There were people who came in public places like markets to alert women about the next vaccination.”* (Centre-Est, vaccinated group)

Some caregivers mentioned their desire for in-person discussions with healthcare workers and community health workers. Written materials were not perceived as helpful because they require a high level of literacy.

*“It is [a] dialogue that is more effective. The posters are for those who went to school. But with dialogue even those who are not educated come to understand.”* (Centre-Est, vaccinated group)

We identified caregivers in both the vaccinated and unvaccinated groups who said they had not received information from any source regarding the 15–18-month visit for MACV. Their lack of awareness was often linked to not being informed at healthcare centers to return for vaccination when the child is 15 months old, inadequate community engagement, and difficulty understanding the vaccination information provided (e.g., print materials targeting illiterate caregivers).

*“It is not all the nurses who say it [reminders about the second year of life vaccines] after a consultation. […] Often it is in the queue that women give information about the new vaccination [to each other while they wait].”* (Centre, vaccinated group)

*“They [health workers] did not tell me anything. It was my first child, and I went to the weighing at the 15th-month visit. They told me that the child must be vaccinated, otherwise I did not know it.”* (Centre-Est, vaccinated group)

### 3.2 Motivations to seek MACV for the child

#### 3.2.1 Awareness

Awareness of the childhood vaccination schedule motivated caregivers to take their children for MACV. When asked about future intention to vaccinate, caregivers with unvaccinated children cited that knowing when to attend vaccination visits and knowing that the vaccines are free would motivate them to vaccinate their children in the future.

#### 3.2.2 Trusted messengers

Getting immunization information from trusted messengers motivated caregivers to get their children vaccinated. We found that healthcare workers were most commonly viewed as trusted messengers by participants across all regions, though a minority of caregivers reported distrust of healthcare workers. In numerous instances, caregivers cited healthcare workers’ recommendations as the driving reason for vaccinating their children.

*“The health workers are there for that. It is their job, and if they say to do something, the mothers will do it for the health of their children. They have more confidence in health workers.”* (Centre, vaccinated group)

*“If the health officials engage women well, those who did not want to go will go.”* (Centre, vaccinated group)

FGDs revealed that caregivers considered other women in the community, including elder village women as key trusted resource persons to help build trust in MACV.

*“Me, I think that it is the fact that women counsel each other. At this time, if women receive the information, they will go to the vaccination.”* (Centre-Est, unvaccinated group)

#### 3.2.3 Perceived benefits of MACV

Caregivers of vaccinated children stated that the availability of MACV at the 15–18-month visit motivated many of them to attend. MACV was perceived as an effective defense against meningitis, which was viewed as a serious and severe health threat. Caregivers also felt that there were no meningitis cases in their communities because of MACV.

*“It’s because of the meningitis vaccination that I went to the 15-month visit. But if there wasn’t the meningitis vaccination, I would not have gone. I went to protect my child against meningitis.”* (Cascades, vaccinated group)

*“We thank God because meningitis no longer attacks our children. I think this is a good thing to have introduced.”* (Nord, unvaccinated group)

### 3.3 Barriers to the uptake of MACV and MCV2

#### 3.3.1 Unavailability or non-provision of vaccines

The lack of availability of the MACV vaccine at the healthcare centers was a recurrent barrier, negatively influencing MACV vaccine uptake. This barrier was consistently reported across all regions and in both vaccinated and unvaccinated FGDs. In some cases, the unavailability was due to providers stating that the number of children at the center would not justify opening a 10-dose vaccine vial. Caregivers complained that failure to make vaccines available consistently meant that they had to embark on multiple costly and time-consuming trips to healthcare centers.

*“It’s an expensive vaccine [in private healthcare centers]. If you want to wait for the government’s [free vaccine], you cannot have it because it is not available.”* (Centre, unvaccinated group)

*“I asked why they only administered one vaccine, and they told me that there was no more vaccine and they would call us when the vaccine was available. But until now, we have not been called.”* (Centre-Est, unvaccinated group)

*“If you travel to go to the hospital, they tell you that there are not a lot of people and to return. If you take the trip more than four times, you can become disappointed. This is what causes many to not get the vaccination.”* (Centre, unvaccinated group)

#### 3.3.2 Knowledge gaps

Respondents noted that there was a lack of knowledge and a lack of communication about the importance of the 15–18-month vaccination visit in the community. Some expressed that vaccination is not well explained to caregivers. Moreover, provided immunization information was perceived as too technical. The lack of knowledge regarding the importance of the vaccine and its benefits was tied non-vaccination.

*“It’s because there are mothers who do not know the importance of vaccination. They do not know the role that vaccination can play. Those who know the importance of vaccination will go with their children [to get vaccinated].”* (Cascades, vaccinated group)

Regardless of their children’s vaccination status, numerous caregivers from different regions expressed that they did not know there is a scheduled vaccination in the second year of the child’s life.

*“They didn’t tell me anything. They took my child to give the vaccine and told me I could leave. They did not tell me to come back because there was a missing vaccination. For me [as far as I was concerned], my child received all his vaccinations, and the vaccinations for my child were completed”* (Centre, unvaccinated group)

#### 3.3.3 Practical constraints

The lack of time to travel to and wait at healthcare centers was discussed in all FGDs. Caregivers reported that traveling to healthcare centers could be difficult at times due to inclement weather and long distances. Moreover, prioritizing livelihood activities during harvest season was cited as a reason for some caregivers missing the scheduled vaccination visits.

*“We know that vaccinating children is a good thing that really helps us, but we often do not have the chance to be at the session.”* (Centre-Est, unvaccinated group)

#### 3.3.4 Past negative experiences

Past unpleasant experiences with healthcare workers for routine immunization visits more pronouncedly emerged as a demotivating factor in seeking MACV among caregivers with unvaccinated children. Some participants reported rude and condescending healthcare worker behavior towards patients and caregivers, including shaming women who have missed visits and who have not adhered to birth-spacing recommendations

*“You spend your time to go, and they yell at you as if you are a child who has never seen the sunrise.”* (Centre, unvaccinated group)

*“There are women who may fall pregnant before the* [last child] *is 15 months. She can feel ashamed, which will discourage her from going to vaccinate her child.”* (Centre-Est, vaccinated group)

Moreover, dissatisfaction with how healthcare workers interacted with caregivers during routine care (non-immunization visits) may also discourage general health-seeking behaviors.

*“They do not treat the children according to the severity of their illness. You often bring your child with a high fever to the center as an emergency and they tell you to get in line. All this means that, unless you are having an extreme emergency, you do not want to go to the healthcare center.”* (Centre, unvaccinated group)

#### 3.3.5 Sociocultural influences

Women who excuse themselves from domestic or agricultural duties to seek vaccination for their children were reportedly viewed as lazy or unproductive by their husbands and the community; this perception contributed to untimely vaccination or missing vaccination sessions. Caregivers also mentioned women needing approval from husbands to take their child for vaccination, which reportedly prevented some women from seeking services.

*“Indeed, when the child’s 15 months arrives during the agricultural season, it is very difficult for us women to have permission to go to the health center […]. Our husbands think we are lazy and that we want to go to the health center to avoid working.”* (Centre-Est, unvaccinated group)

*“There are women who wait for the permission of their husbands before going [ to the health center]."* (Nord, vaccinated group)

#### 3.3.6 Misinformation

Misinformation about vaccination coming from the community and other caregivers was reported across groups, but more pronouncedly among participants in Centre region. Examples of misinformation that emerged were that: 1) children do not need MACV if they have received other vaccines, 2) vaccines given at 9 and 15 months are the same so there is no need to repeat, 3) healthy children do not need to be vaccinated, and 4) vaccines can paralyze, sterilize, or kill children.

*“During a vaccination session, I heard an old man say not to vaccinate our children because the goal of vaccination is to make the child sterile.”* (Centre, vaccinated group)

## 4 Discussion

The qualitative findings from caregivers in Burkina Faso revealed the diversity of immunization information and sources, caregiver motivations to seek both MACV and MCV2 for their children, and barriers to vaccinating their children or causing delays in vaccination. Although we uncovered that caregivers received immunization information from multiple and diverse sources, healthcare workers were the most common trusted source of information. The recommendation of healthcare workers was cited as a motivating factor for returning with the child for the 15–18-month visit where children received MACV and MCV2. Other motivating factors included awareness of the routine vaccination schedule and awareness of the benefits of MACV in preventing meningitis, which was viewed as a serious health threat. Caregivers also expressed a range of barriers that prevented or caused delays in seeking vaccination services for their children. Caregivers were discouraged by the unavailability of the vaccines, which were often linked to the withholding of vaccines by healthcare workers possibly due to a misguided concern about vaccine wastage. Other recurring barriers included knowledge gaps about the need for the 15–18-month visit, practical constraints, past negative experiences with healthcare workers, sociocultural influences such as stigmatizing attitudes towards women who leave home to take their children for vaccination, and misinformation about MACV, MCV2, and vaccines in general.

Findings from the FGDs were consistent with the Health Belief Model, in which perceived threat (susceptibility and severity) of a disease is an important driver of adopting protective behaviors [21], including vaccination [22], [23], [24]. Given the reported high awareness of the likelihood and severity of meningitis among the population in Burkina Faso, there was an a priori expectation that this perceived threat would greatly motivate caregivers to seek MACV for their children despite the vaccine being offered in the second year of life. Another related expectation was that MACV introduction would help improve vaccination coverage for MCV2 since both vaccines are administered concomitantly at the 15–18-month visit; observed increases in MCV2 coverage after MACV introduction were significant but could not be distinguished from expected increases over time [9]. Beyond Burkina Faso, a 2012 systematic review found no evidence of new vaccine introductions having an impact (positive or negative) on the coverage of existing vaccines offered in the routine childhood immunization schedule [25]. Case studies in six LMICs found that administrative coverage of routine vaccines remained unchanged after new vaccine introductions despite immunization staff’s reported perception of increased coverage during key informant interviews [26].

The barriers captured in our assessment may help explain why meaningful coverage improvements have not been observed following vaccine introductions in Burkina Faso and other LMICs. A major barrier cited by caregivers was the need to return multiple times to healthcare centers because healthcare workers often withheld the vaccine until there were enough eligible children present. This may be explained by a reluctance of healthcare workers to open multidose vaccine vials to avoid wastage, a behavior documented both in Burkina Faso [19] and elsewhere [27], contrary to WHO vaccine management guidelines [19]. Moreover, we found misinformation among caregivers regarding the need for MCV2. Some caregivers expressed that since the vaccines given at 9 and 15 months are the same, there was no need for the 15-month visit. This finding reflects gaps in knowledge about measles vaccine being a 2-dose series, coupled with poor awareness of MACV being offered at the 15–18-month visit despite comprehension of the risk and severity of meningitis. These findings emphasize the need to provide reminders and simple-to-understand information about the vaccination schedule overall, especially for vaccines offered in the second year of life that may have lower awareness among caregivers.

Our results are consistent with data from high-income countries, showing that healthcare worker recommendations are a strong predictor of vaccination uptake [28], [29], [30]. In our assessment, healthcare workers were viewed as trusted sources of information and their recommendations motivated caregivers to return to the healthcare center for scheduled vaccination visits. Healthcare workers, including community health workers, should be leveraged as important communicators to build and strengthen confidence in vaccines and the vaccination process. Training healthcare workers on motivational interviewing to address vaccination concerns could be a promising technique to improve healthcare workers’ interpersonal communication with caregivers [31], [32]. Given that some caregivers inevitably miss scheduled vaccination visits—hence, missing the receipt of information from healthcare workers—reminders and support for caregivers from the community health workers and other trusted persons from the community are necessary to ensure timely vaccination of children. For example, other caregivers from the community who were viewed as trusted messengers in our assessment may help build trust and confidence in immunization services.

Our findings shed light on programmatic and policy considerations when introducing MACV into the EPI in other countries in the meningitis belt. Countries introducing other new vaccines recommended in the second year of life may face similar challenges. For example, WHO recommends typhoid conjugate vaccine (TCV) introduction into the routine immunization schedule at 9 months of age or in the second year of life [33]. Countries choosing to introduce TCV in the second year of life are most likely to do so at the 15–18- month visit and may face the barriers seen in this study. In addition, malaria vaccine has been introduced in three pilot countries as part of the Malaria Vaccine Implementation Programme as a four-dose schedule, with the final dose administered late in the second year of life at 22 or 24 months of age[34]. The findings we report here may be useful in designing successful strategies to optimize vaccination coverage for these second year of life vaccines, thereby increasing population immunity to these devastating diseases.

These results also have implications for the current COVID-19 pandemic. Global guidance stresses the need to maintain immunization as an essential health service during COVID-19 disruptions [35]. Clear communication from trusted messengers about changes in immunization service delivery due to COVID-19 has been identified as an important component to maintaining health systems in the pandemic context [36], [37]. Our study has identified that caregivers of young children value face-to-face communication from healthcare workers to receive information on vaccination for their children. This highlights the need to continually support healthcare workers in clearly communicating with caregivers on the availability, efficacy, and safety of vaccination, and the severity of vaccine-preventable diseases. Identifying trusted messengers and preferred methods of communication is also crucial for developing strong strategies to promote the acceptance and uptake of COVID-19 vaccines [36].

## 5 Limitations

FGDs were conducted in local languages and later transcribed into French. Coding was done on the French version of the transcripts. Subsequently, in our analysis, we translated coded excerpts from the French transcripts into English. Some loss of meaning may have occurred during the iterative translations. Additionally, because only four districts were sampled for this assessment, these results may not be generalizable to the general population of caregivers in Burkina Faso. However, it should be noted that the aim of qualitative assessments is not generalizability but to capture subtleties and perceptions around a topic that are not easily quantifiable. The underlying meaning of the themes identified in our assessment may hold transferability to other settings in Burkina Faso and elsewhere. In addition, the sampling strategy resulted in FGDs comprised exclusively of mothers of MACV-eligible children; therefore, perspectives of fathers and other community stakeholders are not captured in these results.

## 6 Conclusions

To the best of our knowledge, our assessment was the first to examine caregivers’ perceptions about the newly introduced MACV into the routine childhood immunization schedule at 15–18 months of age in Burkina Faso or anywhere else. The findings from our assessment add a new understanding, in addition to reinforcing the existing understanding of motivations and barriers to seeking immunization services in the second year of life. The findings point to the importance of strong communication efforts by healthcare workers and other trusted sources of information regarding the timing and benefits of the new vaccines, especially after 11 months of age. Improving MACV and MCV2 coverage may require increased investments in community engagement to improve knowledge of immunization visits in the second year of life. Additionally, training healthcare workers on open vial policies may help prevent missed opportunities for vaccination. New vaccine introductions should be accompanied by community assessments to understand the dynamics around information (and misinformation), motivations, and barriers related to uptake of the new vaccine.

## 7 Disclaimer

The findings and conclusions in this report are those of the authors and do not necessarily represent the official position of the Centers for Disease Control and Prevention or UNICEF.

## CRediT authorship contribution statement

**Brooke Aksnes:** Formal analysis, Writing – original draft, Writing – review & editing, Visualization. **Jenny A. Walldorf:** Writing – original draft, Writing – review & editing, Conceptualization, Methodology, Supervision, Project administration, Funding acquisition. **Sylvain F. Nkwenkeu:** Formal analysis, Conceptualization, Methodology, Investigation. **Robert L. Zoma:** Methodology, Formal analysis, Investigation. **Imran Mirza:** Writing – review & editing, Conceptualization, Methodology, Supervision, Funding acquisition. **Felix Tarbangdo:** Formal analysis, Investigation. **Soukeynatou Fall:** Methodology, Supervision. **Sansan Hien:** Methodology, Supervision. **Cesaire Ky:** Investigation. **Ludovic Kambou:** Conceptualization, Project administration. **Alpha Oumar Diallo:** Methodology, Project administration. **Flavien H. Aké:** Project administration, Funding acquisition. **Cynthia Hatcher:** Conceptualization, Writing – review & editing, Project administration, Funding acquisition. **Jaymin C. Patel:** Writing – review & editing. **Ryan T. Novak:** Conceptualization, Writing – review & editing, Project administration, Funding acquisition. **Terri B. Hyde:** Conceptualization, Writing – review & editing, Project administration, Funding acquisition. **Isaïe Medah:** Conceptualization, Project administration. **Heidi M. Soeters:** Writing – original draft, Writing – review & editing, Conceptualization, Methodology, Supervision, Project administration. **Mohamed F. Jalloh:** Formal analysis, Writing – original draft, Writing – review & editing, Conceptualization, Methodology, Supervision.

## Declaration of Competing Interest

The authors declare that they have no known competing financial interests or personal relationships that could have appeared to influence the work reported in this paper.
