# Supplementary material for: Vaccination information, motivations, and barriers in the context of meningococcal serogroup A conjugate vaccine introduction: A qualitative assessment among caregivers in Burkina Faso, 2018
Source: Vaccine. Author manuscript; Available in PMC 2022 Oct 15. (PMC8519392; doi:10.1016/j.vaccine.2021.09.038)
Supplement: Supplemental Material [file NIHMS1748112-supplement-Supplemental_Material.docx]

SUPPLEMENTAL MATERIAL

**Guide for focus group discussions with caregivers of MACV eligible children**

**Version A (child was vaccinated with MACV)**

*Note: Moderator should assign numbers for each person before the discussion begins*

INTRO & OPENING STATEMENT

Thank you for taking the time to join us for this focus group discussion. I am ________, your facilitator, and this is ______ who will be taking notes as we talk, and we will also be recording.

We hope for frank and open discussion where there are no right or wrong answers. Everyone’s perspective is valued. The discussion will take around 1 hour. Do you have any questions?

**BARRIERS/ACCESS**

- - - 1. Let’s talk about healthcare for your children. What are the most common health problems for which people seek care for their children?
  1. Where do you usually seek healthcare for your children?
     1. Probe: From a traditional or religious healer? In a health facility? Government or private facility? Where do you go first? How do you decide where to seek care/help?
     2. Probe: How is this decision influenced or not influenced by the illness?
     3. Probe: If a child gets sick and needs treatment, where do parents in this community go first?
  2. If a parent wants to prevent their child from getting sick, what are some of the things they do?

Perceptions of meningitis and measles

1. What are people’s general understanding of meningitis and measles in this community? (*probe separately for each disease*)
   1. Probe: How much of a problem is it?
   2. Probe: Who does it affect the most, and how severe?
   3. Probe: What is usually done if it is suspected?
   4. Probe: How can it be prevented?
2. In this community, how much of a problem are meningitis and measles among young children under the age of 2 years? (*probe separately for each disease*)
3. Probe: What do people think is the cause of the disease in young children?
4. Probe: How would you know if a child has it?
5. Probe: What is usually done if a child is suspected of it?
6. Probe: How can the disease be prevented among young children?
   - 1. How might these prevention efforts differ when compared to what is done for older children?

Awareness and information sources

1. How much are people aware of the vaccines offered at the 15-month immunization visit?
2. Probe: How do people learn about this visit, and what were they told?
3. Probe: Before availability of MenA vaccination, what was done to prevent meningitis in young children?
4. Probe: How is MenA vaccination promoted in your community?
   - 1. Which vaccination promotion methods do you think are the most effective, and for what reasons?

Perceptions of MenA vaccination introduction

1. Please tell us about your views, thoughts, and feelings about the introduction of MenA vaccination now offered at the 15-month visit.
2. Probe: How important do you think it is for children in this community, compared to other routine vaccinations for children?
3. Probe: Overall, what are people saying about it in the community?
4. Probe: What concerns or questions do you or others in the community have about it?

Motivations

1. What motivated you to bring your child to the 15-month immunization visit?
2. Probe: What are your top three reasons for bringing in the child?
3. Probe: If not for MenA vaccination, would you still have brought the child for RR2 at the 15-month visit? Please say more about this.
4. Probe: What else would motivate you or other caregivers to bring your children for the 15-month vaccination visit?

Barriers

1. In general, what prevents some caregivers from bringing their children to the health facility for vaccination?
2. Probe: Are the reasons the same for MenA and RR2 vaccination? Please help us understand.
3. Probe: What can be done to encourage these caregivers to bring in their children for the 15-month visit for MenA and RR2?
4. Probe: Who would they trust to talk to them about the MenA and RR2 vaccines offered at the 15-month visit, and why?
5. What will be your suggestion for helping children receive all their recommended vaccines according to the schedule?

CLOSING STATEMENT

We have now reached the end of our discussion. We would like to thank you for participating in today’s discussion. The information you shared are very important and will help us better understand how to improve immunization services for children in your district.

**Version B (child was not vaccinated with MACV)**

*Note: Moderator should assign numbers for each person before the discussion begins.*

INTRO & OPENING STATEMENT

Thank you for taking the time to join us for this focus group discussion. I am ________, your moderator, and this is ______ who will be taking notes as we talk, and we will also be recording.

We hope for frank and open discussion where there are no right or wrong answers, everyone’s perspective is valued. The discussion will take around 1 hour. Do you have any questions?

**Repeat questions 1-4 from Version A**

Perceptions of MenA vaccination

1. For those of you who are aware of MenA vaccination now offered at the 15-month visit in this community, please tell us more about your views, thoughts, and feelings about it?
   1. Probe: How important do you think it is for children in this community, compared to other routine vaccinations for children?
   2. Probe: Overall, what are people saying about it in the community?
   3. Probe: What concerns or questions do you or others in the community have about it?

Barriers

1. What are the main reasons why you have not taken your child to the health facility for the 15-month vaccination visit?
2. Probe: Has distance to the health facility influence your decision? If so, how?
3. Probe: Has cost or payment influence your decision? If so, how?
4. Probe: Has your trust in immunization staff influence your decision? If so, how?
5. Probe: Are there religious or cultural reasons for not going? If so, please say more.
6. Probe: Are there any other concerns you have? If so, please tell us more.
7. Have you had any other instances in the past in addition to the 15 month visit when you did not take your child for a scheduled vaccination?
   1. Probe: Did you eventually take the child? Please say more about your decision.
   2. Probe: How did that experience influence your decision to not attend the 15-month visit (for MenA and RR2) vaccination this time around?

Motivations

1. What can be done to encourage you to take your child for the 15-month visit for MenA and RR2 vaccination?
2. Probe: Of the things you’ve mentioned, which are the top 3 most important to you?
3. Probe: Who would you trust the most to talk to you and what would you need to hear from them to help you decide to take your child to the 15-month visit for MenA and RR2 vaccination?
4. What will be your suggestion for helping children receive all their recommended vaccines according to the schedule?

CLOSING STATEMENT

We have now reached the end of our discussion. We would like to thank you for participating in today’s discussion. The information you shared is very important and will help us better understand how to improve immunization services for children in your district.
